# Modeling stand biomass for Moso bamboo forests in Eastern China

**DOI:** 10.3389/fpls.2023.1186250

**Published:** 2023-07-27

**Authors:** Xiao Zhou, Zixu Yin, Yang Zhou, Xuan Zhang, Ram P. Sharma, Fengying Guan, Shaohui Fan

**Affiliations:** ^1^ International Center for Bamboo and Rattan, Key Laboratory of National Forestry and Grassland Administration, Beijing, China; ^2^ National Location Observation and Research Station of the Bamboo Forest Ecosystem in Yixing, National Forestry and Grassland Administration, Yixing, China; ^3^ Institute of Forestry, Tribhuwan University, Kathmandu, Nepal

**Keywords:** aboveground biomass, belowground biomass, random effect, heteroscedasticity, Moso bamboo

## Abstract

Stand biomass models can be used as basic decision-making tools in forest management planning. The Moso bamboo (*Phyllostachys pubescens*) forest, a major forest system in tropical and subtropical regions, represents a substantial carbon sink, slowing down the rise of greenhouse gas concentrations in the earth’s atmosphere. Bamboo stand biomass models are important for the assessment of the contribution of carbon to the terrestrial ecosystem. We constructed a stand biomass model for Moso bamboo using destructively sampled data from 45 sample plots that were located across the Yixing state-owned farm in Jiangsu Province, China. Among several bamboo stand variables used as predictors in the stand biomass models, mean diameter at breast height (MDBH), mean height (MH), and canopy density (CD) of bamboo contributed significantly to the model. To increase the model’s accuracy, we introduced the effects of bamboo forest block as a random effect into the model through mixed-effects modeling. The mixed-effects model described a large part of stand biomass variation (*R*2 =^ ^0.6987), significantly higher than that of the ordinary least squares regression model (*R*2 =^ ^0.5748). Our results show an increased bamboo stand biomass with increasing MH and CD, confirming our model’s biological logic. The proposed stand biomass model may have important management implications; for example, it can be combined with other bamboo models to estimate bamboo canopy biomass, carbon sequestration, and bamboo biomass at different growth stages.

## Introduction

Forest absorbs a tremendous amount of carbon dioxide from the atmosphere through photosynthesis, accumulates biomass in the stem, branches, leaves, and roots, and contributes to the organic carbon in the soil (Yen and Lee, 2011; Yen, 2016; Yuen et al., 2017). Forests play an important role in the terrestrial carbon cycle and an irreplaceable role in maintaining the global climate system and slowing down the rise of atmospheric greenhouse gas concentration (Chen et al., 2009; Jyoti Nath et al., 2009; Zachariah et al., 2016). Consequently, many countries around the world have paid much attention to the monitoring and evaluation of forest biomass.

Bamboo forests share a large part of the forest ecosystem in subtropical and tropical regions (Scurlock et al., 2000; Cao et al., 2011; Song et al., 2011; Song et al., 2017). According to the Ninth Forest Inventory of China, bamboo forest covers an area of 6.41 million hectares (about 3.57% of total forest coverage), accounting for approximately a quarter of the global forest coverage (FAO, 2010). Moso bamboo (*Phyllostachys pubescens*) is one of the most important economic species in China. Relative to other woody plants, Moso bamboo has many advantages due to its rapid growth, high yield, and multiple uses (Scurlock et al., 2000; Lu, 2001). In addition, the Moso bamboo forest has the capacity to accumulate large biomass yields in a short growth period (Jyoti Nath et al., 2009; Yen and Lee, 2011; Yang, 2016; Yen, 2016; Zhou et al., 2022a). Moso bamboo forests help slow down the rise of greenhouse gas concentration in the earth’s atmosphere, and therefore quantifying bamboo forest biomass can be a fundamental basis for assessing the contribution of the carbon cycle to terrestrial ecosystems (Zheng et al., 1998; Yue et al., 2018; Zhou et al., 2022b). Currently, many regions are suffering from a wood shortage, and bamboo forests can be an alternative source of wood. This research sought to quantify and model bamboo forest biomass and carbon sequestration in China.

Biomass measurement is both a time- and labor-demanding task because the entire bamboo stem needs to be felled, roots extracted, dried, and weighed for biomass quantification, which is difficult to do practically (Willebrand et al., 1993; Zhou, 2006a; Zeng, 2015; Fu et al., 2016; Zhou et al., 2022a). The biomass models, which are constructed based on sample data acquired from the population of interest, have frequently appeared in modeling forest productivity, nutrient cycling, and carbon sequestration by forest ecosystems. At the same time, methods of constructing biomass models have evolved from a simple least square regression to complex nonlinear mixed-effects modeling and dummy variable modeling (Zeng et al., 2011; Fu et al., 2012; Zeng, 2015; Zhou et al., 2021; Zhou et al., 2022a; Zhou et al., 2022b; Zhou et al., 2022c). Currently, the application of biomass models is increasingly used to estimate the biomass of plant communities.

Biomass models are based on allometric functions (Chen et al., 2009; Zeng et al., 2011; Fu et al., 2012; Zeng, 2015; Fu et al., 2016; Lin et al., 2017). In recent years, research on plant biomass has included (1) building an individual-based biomass model or its application for estimating biomass in a large scale (e.g., biomass of a stand, forest of region, province, or country) (Zhou 2006a, Zhou et al., 2006b; Chen et al., 2009; Zeng et al., 2011; Fu et al., 2012; Zeng, 2015; Lin et al., 2017; Zeng et al., 2017); (2) constructing compatible individual biomass model systems by considering different growth conditions of different components of individual plants (Fu et al., 2016; Yang, 2016; Zhou et al., 2022a); (3) studying biomass or carbon storage of the forests managed with different measures, different forest types, different site types, and different stand densities (Zhou et al., 2006b; Yen and Lee, 2011; Mensah et al., 2016; Yang, 2016; Qin et al., 2017; Castillo et al., 2018); and (4) studying forest stand biomass accumulation using remote sensing data by extracting important stand variables (e.g., tree height, canopy density, crown width, etc.) (Gonzalez de Tanago et al., 2017; Graves et al., 2018). The belowground component (roots) of Moso bamboo plants is typically large and difficult to distinguish because of the extensive spread of rhizomes, typical of uniaxially scattered bamboo species, whereas the aboveground component can be considered a single plant. The propagation and regeneration of bamboo mainly depend on the spread and growth of rhizomes, commonly referred to as whips, and the emergence of shoots (culms). Aboveground bamboo culms are similar to trees, while the belowground component does not represent an individual plant (Zhou, 1998). Consequently, individual-based total biomass models (aboveground + belowground) cannot be constructed in the same way as for trees. However, despite the potential value of biomass models for the precise estimation of the stand biomass of Moso bamboo, no models currently exist.

Bamboo stand biomass differs with stand structure, stand development stage, and other stand features. For biomass modeling, measurements were carried out in bamboo stands with different site conditions to simulate the relationship between stand biomass and variables affecting biomass variation. Biomass data are generally hierarchically structured (multiple sample plots within the same block and multiple blocks within a forest), and therefore the observations are likely to be spatially correlated (Calama and Montero, 2004; Fu et al., 2017; Zhang et al., 2017; Yang et al., 2020). When traditional modeling methods, such as ordinary least squares (OLS) regression, are used to estimate the model parameters from such a hierarchically structured dataset, an invalid hypothesis test is needed (West et al., 1984; Yang et al., 2020). Mixed-effects modeling is a solution to the problem of correlation among the observations within the same subject (block or sample plot). Mixed-effects modeling takes into account the randomness and stochasticity in the data and thus substantially improves the prediction accuracy of the resulting models (Fu et al., 2017; Pan et al., 2020; Yang et al., 2020; Zhou et al., 2021; Zhou et al., 2022c). A stand-level mixed-effects biomass model is necessary for the precise estimation of Moso bamboo stand biomass and carbon sequestration.

To help overcome the problems resulting from the hierarchical data structure and the absence of bamboo stand biomass estimation models, this study aims to (1) construct a stand biomass model for the Moso bamboo forest using mixed-effects modeling and (2) evaluate the important factors affecting Moso bamboo stand biomass. The presented model will be used for the estimation of Moso bamboo stand biomass and potentially become a reliable tool for carbon accounting and support for bamboo forest management.

## Materials and methods

### Study area

**Figure 1:**
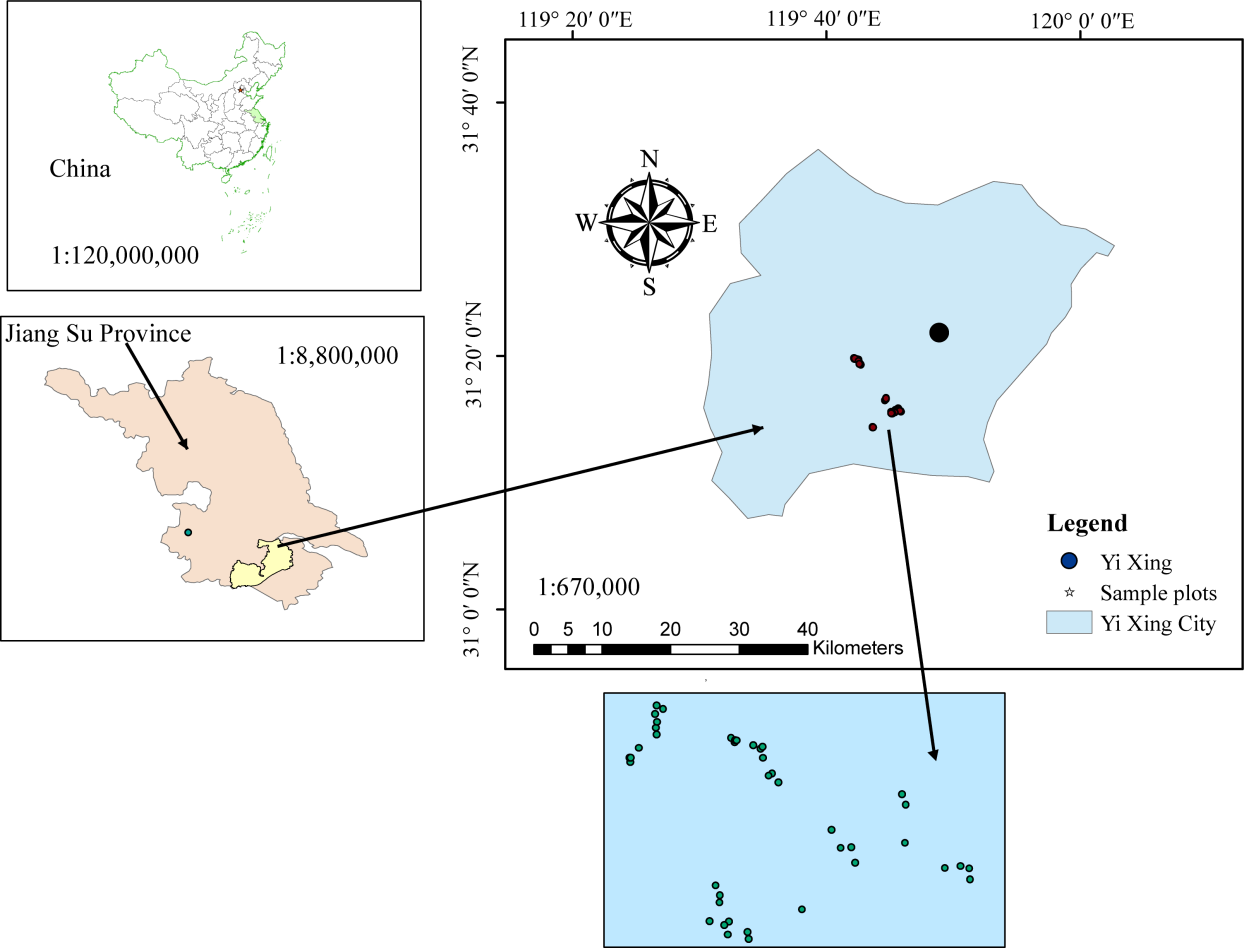
Location (upper left) of the study area: Yixing state-owned forest farm (upper right) in east China, and spatial distribution of five blocks and 45 sample plots (bottom).

This study was conducted using data from Yixing’s state-owned forest farm located in Wuxi City, Jiangsu Province, China (**Figure 1**). The area has a mid-subtropical marine monsoon climate where the annual average maximum temperature is 20°C–24°C and the annual average minimum temperature is 12°C–14° C. Total area of the forest farm is 3,273 ha, including 3,191 ha of forest. There is an estimated 42 million ha of Moso bamboo (*Phyllostachys pubescens*) forest, representing one-third of the total forest area in Jiangsu Province (Huang, 2021).

The traditional management practices employed in the Moso bamboo forests of Jiangsu Province include harvesting mature bamboo culms, winter and spring shoots, shrubs and grass, and tourism activities. Yixing forest farm does not fertilize Moso bamboo forests, but human disturbance to the bamboo forest does occur. The stand density of Moso bamboo is reported to be 2,000–4,000 plants/hm^2^, with a mean DBH of approximately 9.8 cm and an age structure expressed in du of 3:4:3 for I du, II du, and III du, respectively (1-year-old bamboo culms are referred to as 1 du, 2–3 years as 2 du, and 4–5 years as 3 du).

### Data collection

**Figure 2:**
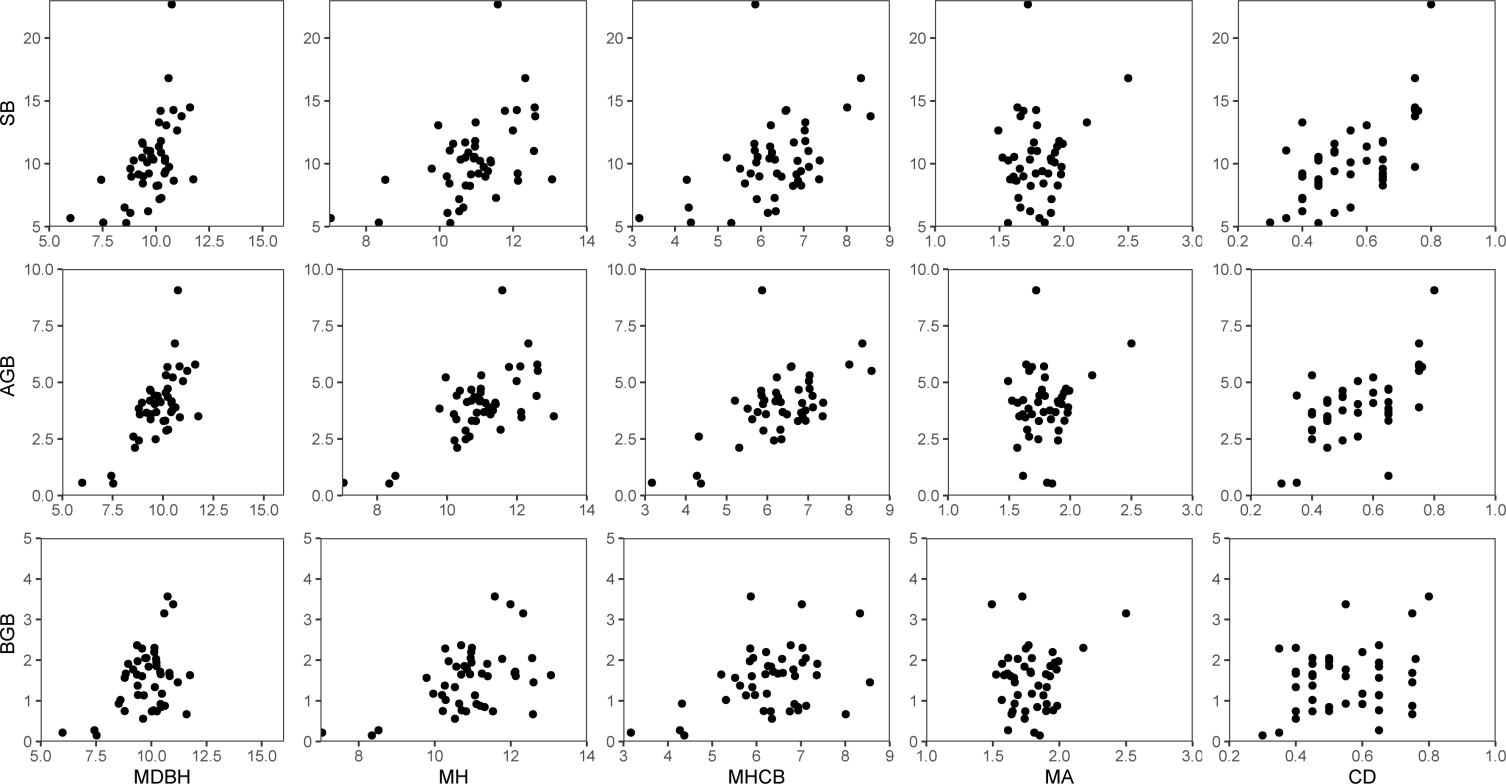
Relationship of stand biomass (SB), total aboveground biomass (AGB), and total belowground biomass (BGB) with each of the five stand variables: mean diameter at breast height (MDBH), mean total tree height (MH), mean height-to-crown base (MHCB), mean age (A), and canopy density (CD).

Data from 45 temporary sample plots established across Moso bamboo forests in 2022 (**Figure 1**) were used. The sample plot size was 20 × 20 m, nested within bamboo forest blocks; altogether, 45 sample plots were nested within five blocks. Blocks were based on different slopes, aspects, and positions of bamboo stands. Plots were positioned randomly to represent bamboo stands with different site conditions. Sample plots were established in stands not suffering considerable damage due to disease, pests, and other factors. Selected sample plots were assumed to provide representative information for the varieties of stand structure and density, bamboo stand height and age, and site productivity. Destructive sampling and data collection were carried out by the International Center for Bamboo and Rattan (ICBR). Within each sample plot, all the standing living bamboo stems with a diameter at breast height (DBH) > 5 cm were measured for DBH, height (H), and height-to-crown base (HCB). Because of the unique growth characteristics of Moso bamboo forests, which involve a vegetative cycle of 2 years (on- and off-year), stand age was expressed as “du” (Tang et al., 2016). One “du” (I) represents 1–2 years, and 2 and 3 “du” (II and III) correspond to 3–4 and 5–6 years, respectively (Tang et al., 2016). The canopy density (CD) of the bamboo forest was determined using a digital camera with a fish-eye lens to take vertical snapshots of the forest canopy between 8:00 and 10:00 a.m. A total of 10 observations were taken 1.5 m above the ground. The CD of sample plots was obtained using image analysis (**Table 1**) (Zhou et al., 2022b; Zhou et al., 2022c). **Figure 2** shows the relationship between different variables and stand biomass.

**Table 1:** Summary statistics of Moso bamboo variables measured.

Variables	Min	Max	Mean	SD
*N* (individuals ha ^-1^)	1,500	4,500	2,762.7778	668.3857
MDBH (cm)	5.9892	11.7517	9.7836	1.0686
MH (m)	7.0405	13.0567	10.9323	1.1106
MHCB (m)	3.1676	8.5550	6.3057	1.0106
CD	0.3	0.8	0.5436	0.1310
MA (du)	1.4918	2.5	1.7921	0.1829
MAG (kg)	9.4112	39.9894	22.9000	5.2069
AGB (kg ha^−1^)	348.2150	5,502.5574	2,449.8198	1,489.8548
BGB (kg ha^−1^)	150.3734	3,568	1,524.6638	758.5164
SB (t ha^−1^)	5.2832	22.6764	10.2648	3.1749

min, minimum; max, maximum; SD, standard deviation; MDBH, mean diameter at breast height; MH, mean total height; MHCB, mean height-to-crown base; CD, canopy density; MA, mean age; MAG, mean aboveground biomass; AGB, aboveground biomass; BGB, belowground biomass.

### Estimation of aboveground biomass

Moso bamboo stands were allocated to du groups (I du, II du, and III du). The DBH and age (du) were measured in each block (five blocks), and the mean DBH for each age was determined. Four culms with mean DBH at each age in each block were cut at ground level (5 blocks * 4 culms * 3 du groups). A total of 60 bamboo culms were harvested.

Each culm was sampled, and the weight of each section was determined. Subsamples were taken from the upper, middle, and lower parts of each 2-m section of the bole, and depending on the diameter, 500–1,000 g was sampled after mixing. The standard branch assessment method was adopted for determining bamboo branch biomass (Zhou, 2006a; Zhou et al., 2006b; Yang, 2016). After bamboo stems were divided into 2-m sections, three standard branches were selected according to the average base diameter and length in each section, a fresh weight for each of them was taken, and the mean weight was obtained. The number of live branches in each section was used to obtain the total fresh weight of branches in each section. After mixing 500–1,000 g branch samples were collected and dried to estimate dry matter. To measure leaf biomass the leaves of selected standard branches were removed, fresh weight was obtained, and a 100–200-g subsample was taken for drying. Drying was performed at 85°C until a constant weight was achieved. Drying took 3–4 days for culm samples, 2–3 days for branches, and 1–2 days for leaves. We used the following formula to determine the dry weight of each sample:


(1) $$f(culm,branch,leaf)=\sum_{i=1}^{n}organ_{dry}\frac{organ_{select-wet}}{organ_{total-wet}}$$

where *n* refers to the culm number of divided or standard branches, *organ* refers to different organs (culm, branch, and leaf), *dry* refers to sample dry weight, *select-wet* refers to sample fresh weight, and *total-wet* refers to total fresh weight.

The total dry biomass of individual bamboo culms was obtained by summing the biomass of each organ.

Aboveground bamboo biomass data were used to calibrate the model proposed by Zhou (2006a) to estimate the aboveground biomass of each bamboo culm in combination with other variables, such as DBH and age. The aboveground biomass (AGB) of the individual was estimated using Eq. (2) (Zhou, 2006a). Total aboveground biomass was obtained by upscaling. There was very little litter present in sample plots, and there was no undergrowth present, so neither was included in the study.


(2) $${\text{AGB}}_{ij}=\beta_{1}{\text{DBH}}_{ij}^{\beta_{2}}{\left(\frac{\beta_{3}A_{ij}}{A_{ij}+\beta_{4}}\right)}^{\beta_{5}}$$

where *AGB_ij_* is the aboveground biomass of individual Moso bamboo in the *j* ^th^ stem of the *i* ^th^ sample plot, is *DBH_ij_* the diameter at breast height of individual Moso bamboo in the *j* ^th^ stem of the *i* ^th^ sample plot, and $A_{ij}$ is the bamboo degree at breast height of Moso bamboo in the *j* ^th^ stem of the *i* ^th^ sample plot.

### Estimation of belowground biomass

In November 2022, 1 m^3^ of soil was excavated from the center of each sample plot, and roots were extracted, cleaned, and weighed. This was replicated three times for each sample plot. Root subsamples were taken for drying and estimation of dry matter. Belowground biomass (BGB) for each sample plot was obtained using the following formula:


(3) $${\text{BGB}}_{ij}=\frac{25}{3}\sum_{n=1}^{3}A_{ijn}$$

where $A_{ijn}$ represents the dry weight of belowground biomass in the *n* ^th^ earthwork of the *j* ^th^ sample plot in the *i* ^th^ block, and ${\text{BGB}}_{ij}$ represents the dry weight of the *j* ^th^ sample plot in the *i* ^th^ block.

The formula for obtaining total stand biomass is as below:


(4) $${\text{SB}}_{ij}=25*\sum_{k=1}^{n}{\text{AGB}}_{ij}+{\text{BGB}}_{ij}$$

.where *k* is the number of Moso bamboo culms in each sample plot, and *SB_ij_* is the stand biomass of Moso bamboo in the *j* ^th^ sample plot of the *i* ^th^ block.

### Modeling approach

#### Selecting basic model

We selected three biomass models representing different forms (linear, empirical, and exponential) (**Table 2**) from the literature (Zeng et al., 2011; Fu et al., 2012; Zeng, 2015; Fu et al., 2016; Zhou et al., 2022a) and used them as base models. Diameter at breast height is convenient to measure and strongly correlated with biomass; consequently, we used MDBH as a predictor variable (*X* = MDBH) in our models to identify the best-performing model. Each model was independently fitted to the entire dataset and compared using the standard statistical indicators (Eqs. 5–8).

**Table 2:** SB candidate stand models considered.

Model No.	Model	Model form
**I.1**	${\mathrm{SB}}_{ij}=\beta_{0}+\beta x$	Linear
**I.2**	${\mathrm{SB}}_{ij}=\beta x^{\alpha }$	Empirical
**I.3**	${\mathrm{SB}}_{ij}=\alpha \exp(-\beta x)$	Exponential

$SB_{ij}$ , stand biomass j^th^ sample plot nested in the ith block; x , vector of stand variables; $\beta_{0}$ , β , α , $\alpha_{0}$ , and $\alpha_{1}$ are parameter vectors.


(5) $$\text{MD}=\frac{1}{n}\sum_{i=1}^{n}\left({\text{SB}}_{ij}-\hat{SB_{ij}}\right)$$

(6) $$R^{2}=1-\sum_{i=1}^{n}{\left({\text{SB}}_{ij}-{\hat{\text{SB}}}_{ij}\right)}^{2}/\sum_{i=1}^{n}{\left({\text{SB}}_{ij}-\frac{\sum_{i=1}^{n}{\text{SB}}_{ij}}{n}\right)}^{2}$$

(7) $$RMSE=\sqrt{\frac{1}{n}\sum_{i=1}^{n}{\left({\text{SB}}_{ij}-{\hat{\text{SB}}}_{ij}\right)}^{2}}$$

(8) $$\text{TRE}=\sum_{i=1}^{n}\left|{\text{SB}}_{ij}-{\hat{\text{SB}}}_{ij}\right|/\sum_{i=1}^{n}{\hat{\text{SB}}}_{ij}$$

where ${\mathrm{SB}}_{ij}$ is the stand biomass of the *j* ^th^ sample plot in the *i* ^th^ block, and *n* is the number of sample plots. MD is the mean deviation (residual), RMSE is the root mean square error, TRE is the total relative error, and *R* ^2^ is the coefficient of determination.

#### Additional predictor variables

In addition to MDBH, Moso bamboo biomass could be largely affected by the size and vigor of a stand or individual bamboo, site quality, and stand density or competition (Rijal et al., 2012; Fu et al., 2013; Fu et al., 2017). A total of 13 variables, including seven stand- and individual-level variables and six variables describing site quality (**Table 3**), were evaluated for their potential contributions to the variations of SB using the best model. The random effect of the block was added to the model to reflect site quality.

**Table 3:** Stand-level variables evaluated for stand biomass models.

Effects by group	Variables
Stand or tree size and vigor	Stand density (*N*), canopy density (CD), sample plot arithmetic mean diameter (AMD), sample plot quadratic mean diameter (QMD), sample plot dominant bamboo diameter (DD), sample plot arithmetic mean height (AMH), mean age, mean height-to-crown (MHCB)
Site quality	Sample plot dominant bamboo height (DH), latitude (LE), longitude (LG), aspect (AT), slope (SE), elevation (EN)

We selected the predictor variables to be included in the SB models using graphical analysis and consideration of the correlation statistics of the variables included in the analysis (Uzoh and Oliver, 2008). Moreover, different combinations of stand variables and their transformations were evaluated based on RMSE and Akaike’s information criterion (AIC). The best-performing expanded base model was then used to construct the nonlinear mixed-effects stand biomass model.

#### Nonlinear mixed-effects stand biomass model

The nonlinear mixed-effects stand biomass (NLME SB) model was constructed by introducing block-level random effects into the expanded base model. The NLME model alternatives with all the possible expansion combinations of the fixed-effects parameters with the random effects were fitted to the data, and a model variant with the smallest AIC and the largest log-likelihood (LL) was selected for further analyses. To avoid the problems caused by over-parameterization, we performed the likelihood-ratio test (LRT) (Fang and Bailey, 2001).

Our preliminary analysis showed that spatial correlations had little influence on the SB model, but there was significant heteroscedasticity. We then introduced the variance–covariance matrix (Eq. 9) to reduce heteroscedasticity (Davidian and Giltinan, 2003).


(9) $$R_{i}=\sigma^{2}G_{i}^{0.5}\Gamma_{i}G_{i}^{0.5}$$

where $R_{i}$ is the variance–covariance matrix of the error within sample *i*, $\sigma^{2}$ is a scaling factor of the error dispersion, which is equal to the residual variance of the estimated model (Grégoire et al., 1995), *G_j_* is the diagonal matrix describing heteroscedasticity of the sample plots, and $\Gamma_{i}$ is a matrix describing autocorrelations of the observations within the block, which was not significant. Therefore, $\Gamma_{i}$ was assumed to be an identity matrix.

We evaluated the effectiveness of three commonly used variance functions (Eqs. 10–12) in reducing heteroscedasticity (Fu et al., 2020; Yang et al., 2020), an exponential function, a power function, and a constant plus power function. We added each of the functions to the optimal model that was selected as above, and AIC and Log-likelihood (LL) were used to evaluate the effectiveness of each function.


(10) $$\text{Var}(\xi_{ij})=\sigma^{2}\exp(2\gamma MDBH_{ij})$$

(11) $$\text{Var}(\xi_{ij})=\sigma^{2}{\text{MDBH}}_{ij}^{2\gamma }$$

(12) $$\text{Var}(\xi_{ij})=\sigma^{2}{\left(\gamma_{1}+{\text{MDBH}}_{ij}^{2\gamma_{2}}\right)}^{2}$$

where *MDBH_ij_* is the mean diameter at breast height of the *j* ^th^ sample plot in the *i* ^th^ block, and *γ, γ*_1_, and *γ*_2_ represent the parameters to be estimated.

### NLME SB estimation

The maximum likelihood with the Lindstrom and Bates algorithm implemented in the R software (version 4.1.0) nlme function (Fu et al., 2013; Yang et al., 2020; Zhou et al., 2021) was used to estimate all the NLME model variants. Many studies (Fu et al., 2013; Yang et al., 2020; Zhou et al., 2021) have described Lindstrom and Bates algorithms and nlme functions.

### Model evaluation

The effectiveness of the NLME SB model can be evaluated using independent data. However, additional data acquisition is costly and limited, and therefore, we used the leave-one-out cross-validation (LOOCV) approach because it provides an unbiased error estimate (Fu et al., 2020; Yang et al., 2020; Zhou et al., 2021). The common statistical criteria (Eqs. 5–8) were used to evaluate the prediction performance of the stand biomass model developed in this study.

## Results

### Base model

The best base stand biomass model was selected using four statistical criteria (Eqs. 5–8). Fit statistics from model I.2 showed the smallest RMSE and TRE and the largest *R* ^2^ (**Table 4**), which was ultimately used as a basis for constructing a NLME stand biomass model for Moso bamboo.

**Table 4:** Evaluation statistics of base models.

Model	Parameter estimates		MD	RMSE	TRE	*R* ^2^
	$\beta_{0}$	$\beta_{1}$				
**I.1**	−5.5239 (3.7444)	1.6138^***^ (0.3805)	−4.3027e−15	2.6960	6.5596	0.2949
**I.2**	0.2429 (0.2390)	1.6386^***^ (0.4257)	0.00284	2.6891	6.5239	0.2986
**I.3**	1.9277^*^ (0.8443)	−0.1694^***^ (0.0432)	−0.0035	2.6922	6.5401	0.2969

MD, mean residual; RMSE, root mean square error; TRE, total relative error; R^2^, coefficient of determination.

^***^p< 0.0001; ^**^p< 0.001; ^*^p< 0.05.

### Inclusion of stand covariates

We used only those stand variables that had no collinearity and contributed significantly to the models. In addition to MDBH, other selected variables are MH and CD, which were assumed to describe the stand development stage and stand vigor, respectively. Model I.2, containing MDBH and MH as covariates, showed the smallest RMSE and TRE and the largest *R* ^2^. The final expanded base model (Eq. 13) was then ultimately expanded as a NLME SB model.


(13) $${\text{SB}}_{ij}=\beta_{0}{\text{CD}}_{ij}^{\beta_{1}}{\text{MDBH}}_{ij}^{\beta_{2}}{\text{MH}}_{ij}^{\beta_{3}}(1+\xi_{ij})$$

where *SB_ij_* is the stand biomass of the *j* ^th^ sample plot nested in the *i* ^th^ block; *CD_ij_* is the canopy density of the *j* ^th^ sample plot nested in the *i* ^th^ block, *MDBH_ij_* is the mean diameter at breast height of the *j* ^th^ sample plot nested in the *i* ^th^ block, ${\mathrm{MDBH}}_{ij}$ is the mean height of the *j* ^th^ sample plot nested in the *i* ^th^ block, and *β_0_−β_4_* are model parameters.

### NLME SB model

There were 15 combinations of the random effects with four fixed parameters (*β_0_−β_4_*) of the expanded base model (Eq. 13). All the NLME model combinations converged with the meaningful parameter estimates (**Table 5**). The LRT test suggested that the stand biomass model with parameters $\beta_{1}$ associated with a random effect provided better performance (**Table 6**). Thus, the final NLME SB model was:

**Table 5:** The random effect is associated with a fixed parameter of the expanded base model (Eq. 13) and fit statistics (Eqs. 4–7) of each mixed-effects model variant.

The fixed parameter associated with random effect	MD	RMSE	TRE	*R* ^2^
$\beta_{0}$	0.0182	1.8969	3.1816	0.5748
$\beta_{2}$	0.0213	1.5970	2.2344	0.6987
$\beta_{2}$	−0.0213	1.6379	2.3532	0.6830
$\beta_{2}$	−0.0215	1.6370	2.3506	0.6834
$\beta_{0}$ $\beta_{1}$	−0.0213	1.5969	2.2342	0.6987
$\beta_{0}$	0.0184	1.8969	3.1818	0.5748
$\beta_{0}$	0.0184	1.8969	3.1818	0.5748
$\beta_{1}$	−0.0212	1.5970	2.2345	0.6987
$\beta_{1}$ $\beta_{3}$	−0.0213	1.5970	2.2344	0.6987
$\beta_{2}$	−0.0215	1.6370	2.3506	0.6834
$\beta_{0}$	−0.0213	1.5969	2.2342	0.6987
$\beta_{0}$ $\beta_{1}$ $\beta_{3}$	−0.0213	1.5969	2.2342	0.6987
$\beta_{2}$	0.0184	1.8969	3.1818	0.5748
$\beta_{1}$ $\beta_{2}$ $\beta_{3}$	−0.0213	1.5969	2.2342	0.6987
$\beta_{0}$ $\beta_{1}$ $\beta_{2}$ $\beta_{3}$	−0.0213	1.5969	2.2342	0.6987

MD, mean residual; RMSE, root mean square error; TRE, total relative error; R^2^, coefficient of determination.

**Table 6:** Log-likelihood ratio test (L-ratio) for all the parameter combinations containing random effects.

The fixed parameter associated with random effect	AIC	LL	L-ratio	*p*-value
*β*_1_	193.5683	−90.78417		
$\beta_{0}$ *β*_1_	195.5684	−90.7842	5.5104e−05	0.9941
$\beta_{1}$ $\beta_{2}$	195.5683	−90.78416	1.0082e−05	0.9975
$\beta_{1}$ $\beta_{3}$	195.5683	−90.78417	2.1984e−06	0.9988
*β*_0_ $\beta_{1}$ *β*_2_	197.5684	−90.7842	5.5097e−05	1
$\beta_{0}$ $\beta_{1}$ $\beta_{3}$	197.5684	−90.7842	5.5875e−05	1
$\beta_{1}$ $\beta_{2}$ *β*_3_	197.5683	−90.78417	1.7601e−06	1
$\beta_{0}$ $\beta_{1}$ $\beta_{2}$ $\beta_{3}$	199.5684	−90.7842	5.6052e−05	1

LL, log-likelihood; AIC, Akaike’s information criterion; L-ratio, log-likelihood ratio.


(14) $${\text{SB}}_{ij}=\beta_{0}{\text{CD}}_{ij}^{\beta_{1}}{\text{MDBH}}_{ij}^{\left(\beta_{2}+\mu_{1}\right)}{\text{MH}}_{ij}^{\beta_{3}}(1+\xi_{ij})$$

**Figure 3:**
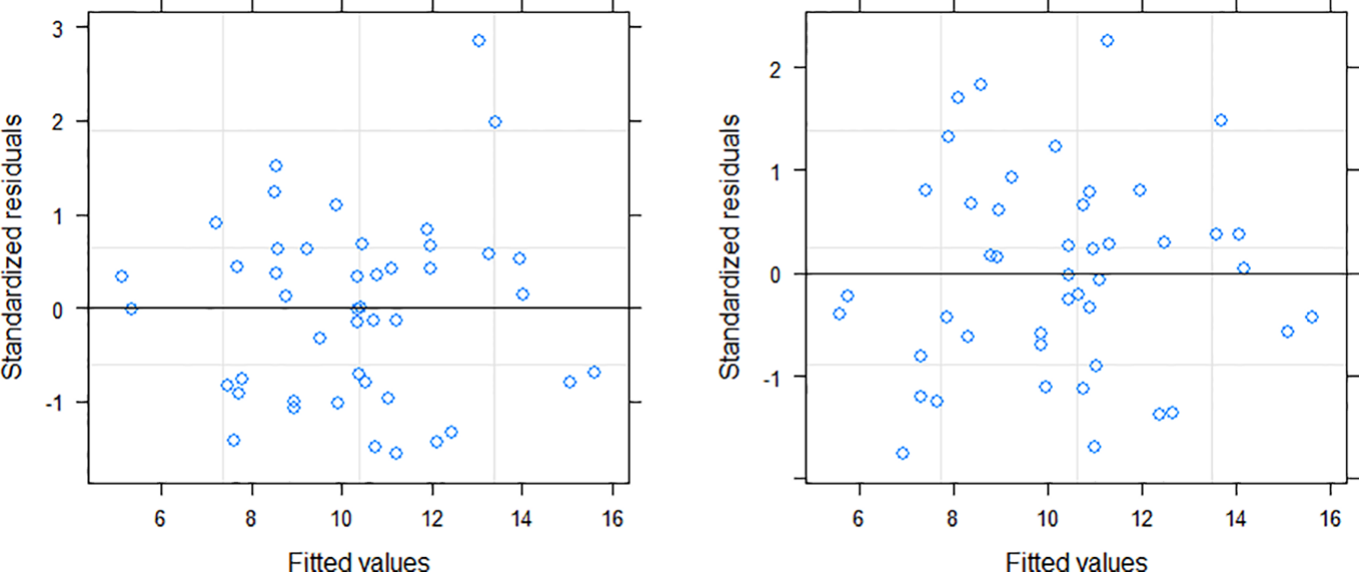
Residuals distribution of the NLME SB model (left: without variance function; right: variance function included).

Among the three variance functions evaluated (Eqs. 10–12), the power exponential form (Eq. 10) applied to MDBH accounted for the variance heteroscedasticity most effectively [**Table 7**; **Figure 3** (right)].

**Table 7:** Comparisons among three variance functions (exponential function, power function, and constant plus power function; Eqs. 10, 11, and 12, respectively) for the NLME SB model.

Variance function	AIC	LL	LRT	*p*-value
None	193.5683	−90.78417		
Equation (9)	187.0951	−86.54754	8.473256	0.0036
Equation (10)	187.544	−86.77198	8.024384	0.0046
Equation (11)	189.5439	−86.77196	8.024412	0.0181

AIC, Akaike’s information criterion, LL, log-likelihood; LRT, likelihood ratio test.

All the parameter estimates obtained for the OLS regression model (Eq. 13) and NLME model (Eq. 14) were significantly different from zero (*p *< 0.05). After the substitution of the estimated parameter values in Eq. (14), the model becomes:


$${\text{SB}}_{ij}=0.1863{\text{CD}}_{ij}^{0.3558}{\text{MDBH}}_{ij}^{1.2123}{\text{MH}}_{ij}^{0.6083}(1+\xi_{ij})$$

where


$$\xi_{ij}∼N(0,1.965)$$

Equation (14) becomes:


$${\text{SB}}_{ij}=0.1271{\text{CD}}_{ij}^{0.1666}{\text{MDBH}}_{ij}^{\left(1.0456+\mu_{1}\right)}{\text{MH}}_{ij}^{0.8902}(1+\xi_{ij})$$

Where


$$\mu_{i}=[\mu_{1}]∼N[0,\hat{\psi }=0.0382]$$

$$\xi_{ij}∼N(0,\hat{R_{ij}}=2.7338{\hat{\text{G}}}_{ij}^{0.5}{\hat{\Gamma }}_{ij}{\hat{\text{G}}}_{ij}^{0.5})$$

${\hat{\text{G}}}_{ij}=\text{diag}[0.0555\text{exp}(0.7043MDBH_{i1}),\ldots 0.0555exp(0.7043MDBH_{in}))$


$$\Gamma_{ij}=I_{ij}$$

**Figure 4:**
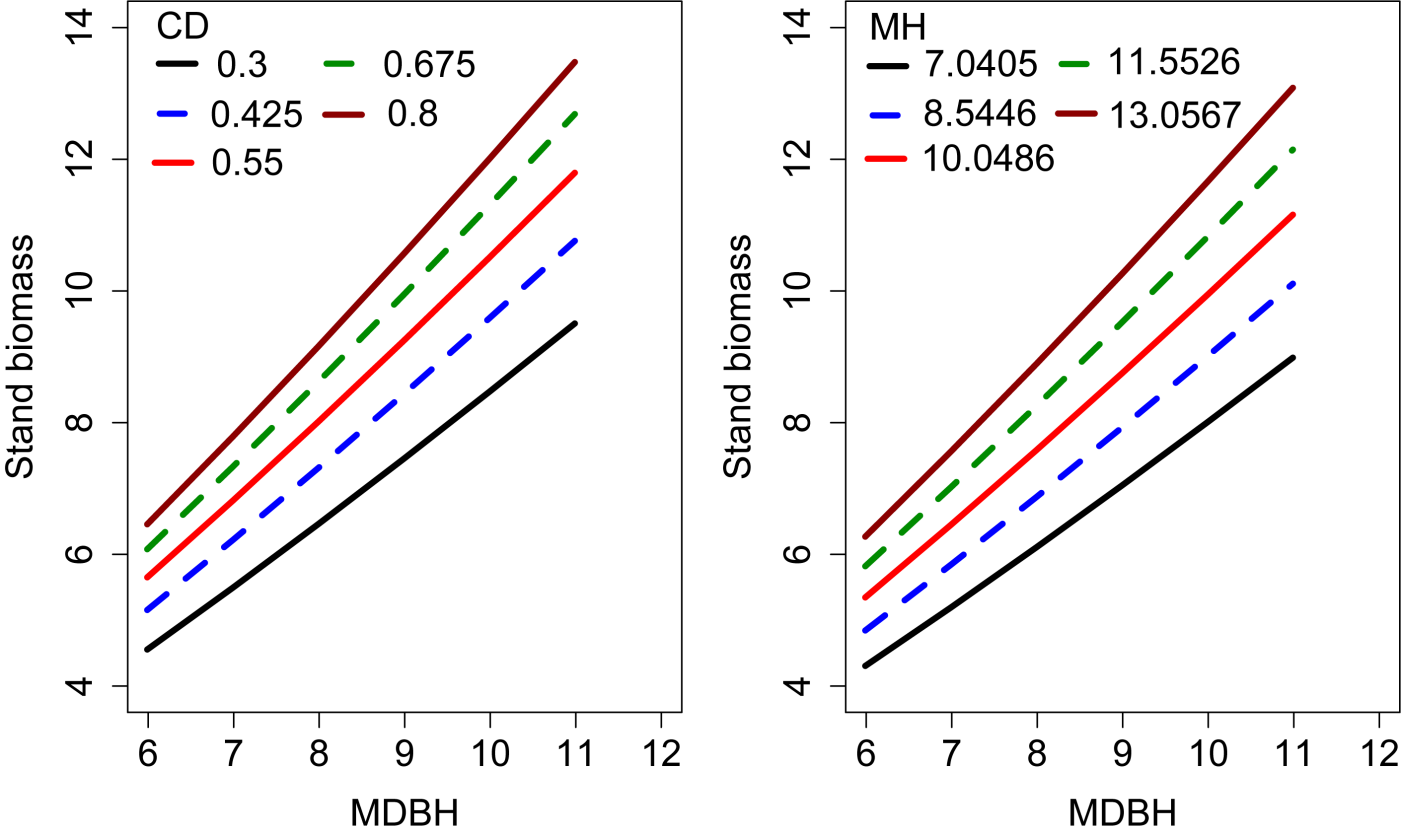
Effects of CD and MH on the stand biomass. The curves were produced using the expanded OLS model (Eq. 13). The mean values of the observed data were used for other variables.

We examined the simulated effects of the predictor variables on the SB (**Figure 4**). This analysis shows each covariate had a significant contribution to the SB variations. The SB increased with increasing MH and CD, which indicates that MH and CD had a significant influence on the SB.

**Figure 5:**
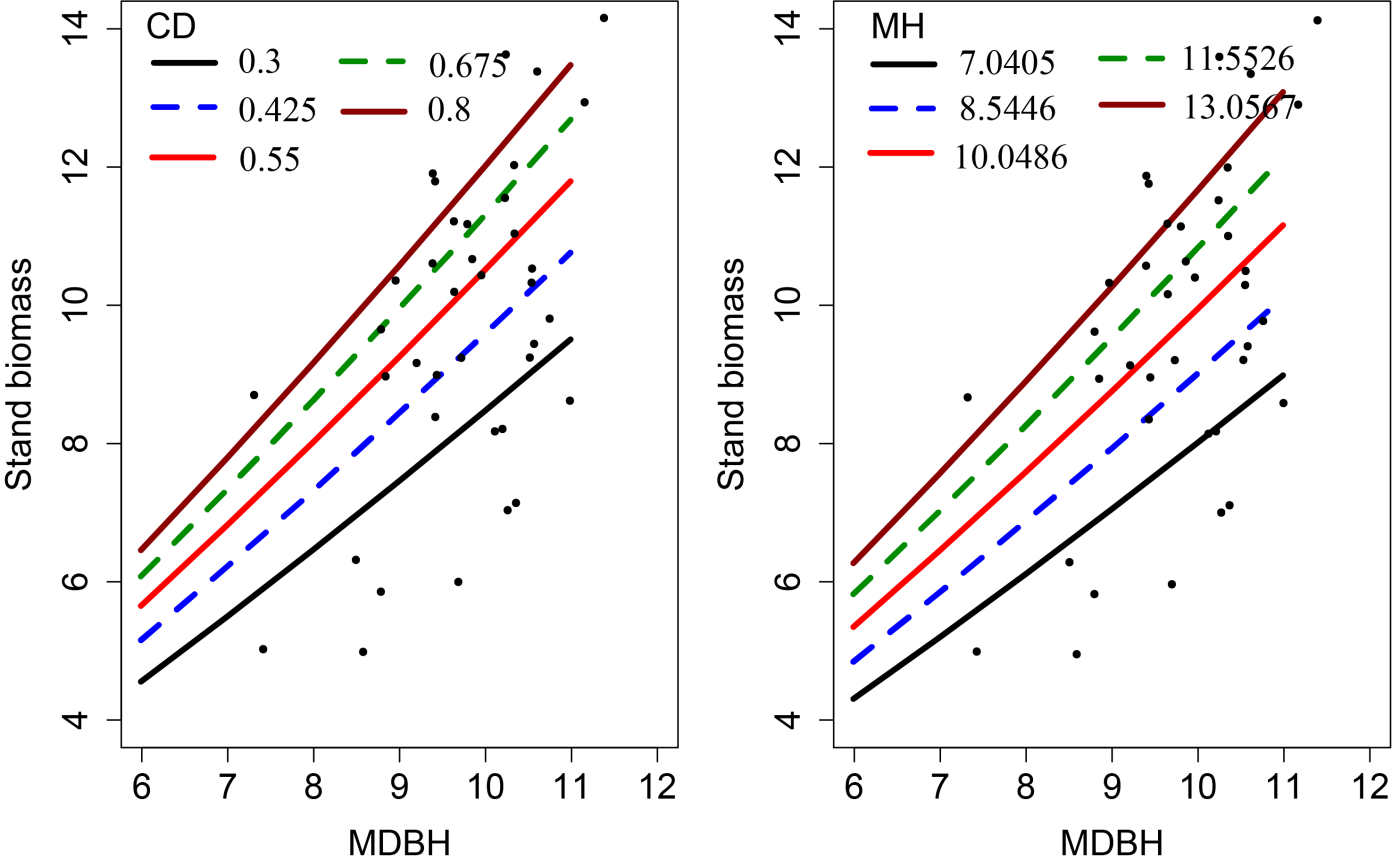
The simulation curves produced with Eq. (13) overlaid on the measured data.

The curves were simulated using Eq. (13) (extended SB model without random effects) passed almost through the middle of the data clouds (**Figure 5**), indicating that the model was biologically plausible and the model parameters could be easily interpreted.

### Model evaluation

**Figure 6:**
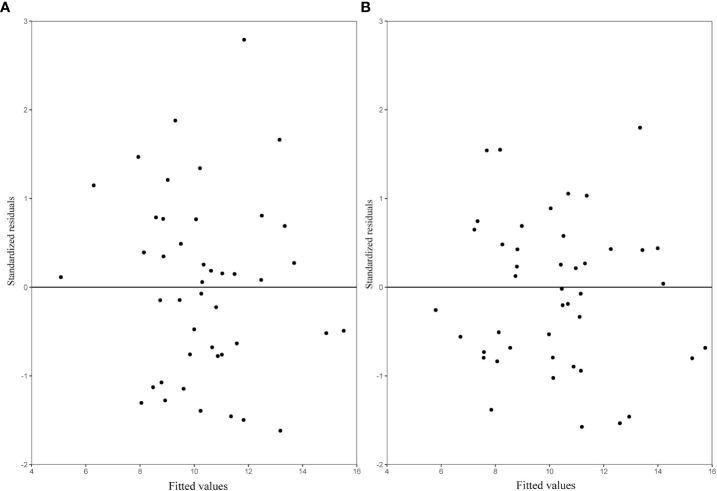
The residuals predicted by leave-one-out cross-validation (LOOCV) for each sample plot **(A)** OLS SB model (Eq. 13); **(B)** NLME SB (Eq. 14), with variance (Eq. 10) included.

We evaluated the expanded base models using the LOOCV approach. The prediction improvement was substantial when the block-level random effect was added to the expanded base model (**Figure 6**). Model validation showed that both the OLS SB model (Eq. 13) and the NLME SB model (Eq. 14) described a large proportion of the variation in stand biomass with no apparent trend in prediction errors (**Figure 6**).

## Discussion

Increasing attention has recently been directed toward the bamboo forest for its numerous advantages over other woody plants. Moso bamboo plays an important role in the global carbon cycle, including the accumulation and storage of carbon that limit the concentration of CO_2_ in the atmosphere (Zachariah et al., 2016; Lin et al., 2017; Xu et al., 2018). There is a lack of methods and models to obtain biomass amount and carbon storage of the bamboo forests, which we have proposed in this study, i.e., a nonlinear mixed-effects stand biomass model for estimating the stand biomass of Moso bamboo. Among several predictor variables evaluated, our model performed the best with three variables (MDBH, MH, and CD) used as predictor variables and one random component added to describe the block-level variations of the bamboo stand biomass. The random block effect included in our model has described site quality and stand competition, effectively improving the model prediction accuracy.

Our models show that stand biomass is significantly related to MDBH and MDC, with the former showing a positive correlation with bamboo stand biomass, indicating MDBH’s promotion of the stand biomass. DBH is an indispensable variable used for biomass estimation and clearly reflects stand vigor (Zeng et al., 2011; Fu et al., 2012; Zeng, 2015; Fu et al., 2016; Zhou et al., 2022a). For a defined bamboo stand density, the larger the MDBH, the greater the biomass of the stand. Some studies have shown that mean bamboo height might be a key index for evaluating the vitality and quality of bamboo (Zhang et al., 2014; Zeng, 2015; Fu et al., 2016; Fu et al., 2017; Pan et al., 2020). Greater MH indicates greater competitiveness and vitality of the forest with relatively high biomass.

Our study also revealed a significant impact of CD on stand biomass (**Figure 4**), as this clearly reflects the site quality and vigor of bamboo forests. However, despite its importance, this was not considered in any of the previous stand biomass modeling studies. The results of this study suggest this approach will be of interest to other researchers working with bamboo forests. The positive relationship between CD and stand biomass reflects higher light interception, higher photosynthesis, transpiration, and other physiological functions (Lawlor, 2009). Interception of solar radiation is a major driver of crown width and DBH growth within a stand (Essery et al., 2008). Better growth and survival of bamboo culms will increase stand biomass.

We also considered site variables that might affect the biomass of Moso bamboo, including slope, slope direction, and slope position. Growth of bamboo in sunny aspects may be better than in shady (Che et al., 2022; Wang et al., 2022). A lower slope angle can also enhance plant growth and biomass accumulation. However, the precision of our model after the inclusion of these variables did not significantly improve. This may be due to the block effect that was included in the model as a random effect, which significantly accounted for the effect of site quality.

Stand density (stems/ha) can significantly affect stand biomass (Zheng et al., 1998; Yue et al., 2018; Zhou et al., 2022b). However, we did not consider stand density in this study, assuming that CD would adequately reflect the degree of stand crowding (Fu et al., 2013; Zhou et al., 2022c). Our analysis showed a greater correlation between CD and SB than between CD and N.

Although adding more variables to a model might improve accuracy to some extent, this can lead to nonconvergence and biased parameter estimation caused by excessive parameterization (Fu et al., 2013; Fu et al., 2017; Zhou et al., 2021), which in this example would increase inventory costs. Consequently, prediction models with the appropriate number of variables are a major concern for forest managers (Calama and Montero, 2005; Adame et al., 2008). We retained three variables in our final stand biomass model to address this concern.

The block-level random effects added to the MDBH predictor provided the best model, as a sense of MDBH difference might have been expressed by the random effect, and the change in MDBH may be closely related to the size of the stand. The application of a NLME stand biomass model after the inclusion of the random effect and the variance function confirmed the model’s promising accuracy. Therefore, we recommend using the NLME SB model (Eq. 13 + Eq. 9) to estimate Moso bamboo stand biomass.

The NLME SB model is suitable for a range of site conditions, including stand density ranging from 1,500 to 4,500 plants/ha, relatively gentle slopes (0°–20°), and a DBH range of 6.0–11.8 cm. While this model has important management and research implications and can help ensure the sustainability of Moso bamboo forests for future generations, it needs to be validated in other stands with similar site conditions.

Our model can assist managers by estimating the biomass of bamboo forests and helping with decisions regarding selective cutting to remove larger stems and retain viable smaller stems. Its use in stands with different site conditions will only be advisable after further research. It may be possible to use remote sensing (Grégoire et al., 2017; Fu et al., 2018; Yang et al., 2020) to assist bamboo stand biomass modeling research while reducing research costs (Askne et al., 2017; Fu et al., 2018).

## Conclusion

Three stand variables strongly correlated with stand biomass were initially incorporated into a least squares regression model for predicting the stand biomass of Moso bamboo. The inclusion of a block-level random effect into a mixed-effects model further improved the predictability of stand biomass. Stand biomass increased with increasing CD and MH, indicating the biological plausibility of the model. This stand biomass model was able to accurately estimate bamboo canopy biomass, carbon sequestration, and biomass at different growth stages, and with further development and validation, it could be a potentially useful decision-aid tool for bamboo forest managers.

## Data availability statement

The original contributions presented in the study are included in the article/supplementary material. Further inquiries can be directed to the corresponding author.

## Author contributions

XiZ, ZY, XuZ, ZL, and FG collected data; XiZ, SF, and FG analyzed data; XiZ, ZL, RS, and FG wrote manuscript and contributed critically to improve the manuscript. All authors contributed to the article and approved the submitted version.
